# Macroeconomic stabilization policy for a dynamic economy: variations on a model by Leitmann and Wan

**DOI:** 10.1007/s10100-025-00978-9

**Published:** 2025-03-31

**Authors:** Reinhard Neck, Dmitri Blueschke, Viktoria Blueschke-Nikolaeva

**Affiliations:** Department of Economics, University of Klagenfurt, Universitätsstrasse 65-67, 9020 Klagenfurt, Austria

**Keywords:** Dynamic games, Feedback Nash equilibrium, Pareto solution, Optimal control, Macroeconomics, Economic policy

## Abstract

In this paper, we modify and extend a model of macroeconomic stabilization policies for a dynamic economy by Leitmann and Wan. We combine a model of long-run output growth and a steady state of no growth with a model of short-run deviations from this growth path based on Keynesian and Monetarist macroeconomic hypotheses. We determine numerically the effects of demand and supply shocks and approximately optimal reactions of fiscal and monetary policies to these shocks for a policy maker in charge of both fiscal and monetary policy using an optimal control algorithm. We also formulate a dynamic game between government (fiscal policy) and central bank (monetary policy) with separate instruments and different preferences for the trade-off between unemployment and inflation and solve it numerically for the noncooperative feedback Nash equilibrium solution and a Pareto optimal solution. The results provide insights into the possibilities of active fiscal and monetary policies and of cooperation and possible conflicts between the institutions responsible for these policies.

## Introduction

In a paper from the 1970 s, Leitmann and Wan Jr. (1979) analyzed optimal stabilization policies for a macroeconomic model, using George Leitmann’s approach to dealing with uncertainty with upper and lower bounds to the uncertain effects of policy instead of stochastic uncertainty. This method has been used since in other papers by Leitmann and co-authors, e.g. Corless and Leitmann (1988). Their elegant analysis emphasizing the stability of the controlled model will not be taken up in the present paper, which concentrates on the specific model by Leitmann and Wan, which, to the best of our knowledge, is the only paper by Leitmann on a macroeconomic topic. Although naturally outdated by theoretical progress made in macroeconomics in the meantime, this model can still be useful as a starting point for policy analysis when taking into account some newer elements of macroeconomics.

In particular, we introduce an expectations-augmented Phillips curve, which is vertical in the long run, as proposed by Friedman (1968) and Phelps (1967) and the related ideas of a natural rate of unemployment and natural (equilibrium) output instead of the simple (long-run) Phillips curve. Moreover, we add a few more short-run feedbacks to the model such as nominal and real market interest rates, a prime rate of the central bank instead of money supply as an instrument and tightness indicator of monetary policy, and a real interest rate. However, we use adaptive expectations instead of the New Classical Macroeconomic hypothesis of rational expectations, which has become a building block also for the New Keynesian Macroeconomic model, e.g., Galì (2008) but which we consider to be unrealistic for a long-run analysis. From Leitmann and Wan, we take over the attractive idea of declining growth towards a long-run equilibrium level of output, interest rate and inflation, which is generally not linked to the short-run shocks that affect the nominal variables and the real interest rate.

The paper is structured as follows. Section 2 describes our model. In Sect. 3, we determine the optimal policies for the model, as in Leitmann and Wan Jr. (1979), under the assumption that both fiscal and monetary policies are under the control of a single political authority. We solve the model without and with a demand and a supply shock using optimal control theory and algorithms for the approximate solution of nonlinear dynamic models under a quadratic objective function. Section 4 analyzes the same model under the assumption of different policy makers for fiscal and monetary policy (government and central bank, respectively), with different instruments and different preferences for the importance of unemployment and inflation. For this, we calculate feedback Nash noncooperative equilibrium and Pareto solutions for the resulting dynamic policy game, which is more realistic than assuming one single decision maker. Section 5 concludes, providing a brief discussion of the policy relevance of our results. We concede that our analysis is much less ambitious than that of Leitmann and Wan as we provide only approximate solutions for the optimization and the game instead of their general theoretical analysis. For practical reasons, we perform our analysis in discrete instead of continuous time and with a finite (but long) time horizon, again in contrast to Leitmann and Wan, and we do not consider uncertainty, although our analysis could be easily extended to include some forms of stochastic uncertainty. An extension to Leitmann’s form of uncertainty remains a question for further research.

## The model

We build a one-country open-economy model (the LWVAR model, standing for the Leitmann-Wan variation). Like Leitmann and Wan Jr. (1979) (p. 111), we assume a dynamic path of real output according to the following function:

1 $$\begin{aligned} \tilde{y}_t= \tilde{y}^{\infty }(1-e^{-\Delta \tau }) \end{aligned}$$

Here $\Delta$ is the assumed parameter defining the path of natural output ($\Delta > 0$ and constant), $\tilde{y}^{\infty }$ is constant and gives the upper bound of the natural output, $\tilde{y}_t$ is the level of natural output for $t \in (0, T)$, and $\tau$ is the time shift parameter for a certain region of the growth process. $\tilde{y}^{\infty } = 100$, $\tau = t + 10$, $\Delta = 0.03$ and $T = 30$^1^ result in $\tilde{y}_T = 69.88$ in the uncontrolled simulation, implying $y_0 = 25.92$, taken here as given, where the values are assumed to be measured in constant price monetary units. Equation (1) implies that the level of natural output, $\tilde{y}_t$, grows by a declining growth rate that approaches 0 as $t\rightarrow \infty$. As with Leitmann and Wan, this assumption guarantees that the model is stable. It can be interpreted to accord with the degrowth idea that, due to the limited resources on planet Earth, every growth in output must eventually come to an end in the infinite future, resulting in a steady state without further growth from then onwards. Analytically, it allows us to combine the growth process with a stable model of deviations from it in the model. We also assume that the policy maker knows the growth process and considers it to be the “ideal” one for the policy problem, that is, it aims at steering the system towards the natural output because otherwise the economic system may become unstable.

The model of short-run deviations from the natural output growth path consists of the following equations:

2 $$\begin{aligned} & \hat{y}_{t}=\delta (\pi ^{ex}_t-\pi _{t})-\gamma \hat{r}_{t} +\rho \hat{y}^{ex}_{t} -\beta \pi _{t}+\kappa \hat{y}_{t-1}-\eta \hat{G}_{t}+ zd_{t}, \end{aligned}$$

3 $$\begin{aligned} & \quad y_{t}=\hat{y}_{t}+ \tilde{y}_{t}, \end{aligned}$$

4 $$\begin{aligned} & \quad r_{t}=i_{t}-\pi _{t}^e, \end{aligned}$$

5 $$\begin{aligned} & \quad \hat{r}_{t}=r_{t}-\tilde{r}_{t}, \end{aligned}$$

6 $$\begin{aligned} & \quad i_{t}=\lambda R_{t} + \zeta i_{t-1} - \chi \frac{\hat{G}_t}{\tilde{y}_{t}}, \end{aligned}$$

7 $$\begin{aligned} & \quad \hat{G}_{t}=G_{t}-\tilde{G}_{t}, \end{aligned}$$

8 $$\begin{aligned} & \quad \hat{R}_{t}=R_{t}-\tilde{R}_{t}, \end{aligned}$$

9 $$\begin{aligned} & \quad \pi _{t}=\pi _{t}^e +\xi \frac{\hat{y}_t}{\tilde{y}_{t}} + \vartheta \pi ^{ex}_t, \end{aligned}$$

10 $$\begin{aligned} & \quad \hat{\pi }_{t}=\pi _{t}-\tilde{\pi }_{t}, \end{aligned}$$

11 $$\begin{aligned} & \quad \pi _{t}^e=\varepsilon \pi _{t-1}+(1-\varepsilon )\pi _{t-1}^e, \hspace{5.0pt}\varepsilon \in [0,1], \end{aligned}$$

12 $$\begin{aligned} & \quad u_{t}=\tilde{u}_t - \omega \frac{ \hat{y}_t}{\tilde{y}_{t}}, \end{aligned}$$

13 $$\begin{aligned} & \quad \hat{u}_{t}=u_t - \tilde{u}_t. \end{aligned}$$

We build a dynamic model consisting of twelve state (endogenous) and two control variables as presented in Table 1. We explicitly model the state variables and their short-term deviations (these are denoted by “$\wedge$”) from the long-run equilibrium. The long-run equilibrium values (these are denoted by “$\sim$”) are assumed to be known by the policy maker(s) and, in the dynamic system, given exogenously. The parameters of the model are explained in Table 2.

**Table 1:** Variables of the LWVAR model with steady-state values $\forall ~ t \in [0,T]$

Name	Value	Description
Control variables		
$G_{t}$	0	Real fiscal surplus
$R_{t}$	0.03	Prime rate
Endogenous variables		
$\hat{y}_{t}$	0	Short-run deviation of output
$y_{t}$	Eq. (1)	Output level
$r_{t}$	0.03	Real interest rate
$\hat{r}_{t}$	0	Short-run deviation of real interest rate
$i_{t}$	0.03	Nominal interest rate
$\hat{G}_{t}$	0	Short-run deviation of real fiscal surplus
$\hat{R}_{t}$	0	Short-run deviation of prime rate
$\pi _{t}$	0	Inflation rate
$\hat{\pi }_{t}$	0	Short-run deviation of inflation rate
$\pi ^{e}_{t}$	0	Expected inflation rate
$u_{t}$	0.03	Unemployment rate
$\hat{u}_{t}$	0	Short-run deviation of unemployment rate
Exogenous variables with steady-state values		
$\tilde{y}_t$	Eq. (1)	Natural output
$\pi ^{ex}_t$	0	Foreign inflation rate
$\hat{y}^{ex}_t$	0	Short-run deviation of foreign output from its natural level
$\tilde{u}_t$	0.03	Natural rate of unemployment
$\tilde{r}_t$	0.03	Steady-state real interest rate
$\tilde{R}_t$	0.03	Steady-state prime rate
$\tilde{G}_t$	0	Steady-state real fiscal surplus
$\tilde{\pi }_t$	0	Steady-state rate of inflation
$zd_{t}$	0	Demand-side shock

**Table 2:** Parameters of the LWVAR model

$\theta$	0.03	Discount rate of policy maker(s)
$\delta$	0.5	Impact on output gap of difference in national and international inflation rates
$\gamma$	0.25	Impact on output gap of deviation of real interest rate from natural rate
$\rho$	0.5	Impact of output gap in foreign country on domestic output gap
$\beta$	0.25	Impact of domestic inflation rate on output gap (size of Pigou effect)
$\kappa$	0.25	Impact of last period’s output gap on current output gap
$\eta$	1	Impact (multiplier) of government budget surplus on output gap
$\lambda$	0.5	Impact of prime rate on nominal interest rate
$\zeta$	0.5	Impact of last period’s nominal interest rate on current nominal interest rate
$\chi$	0.25	Impact of government budget surplus (in relation to potential output) on nominal interest rate
$\xi$	0.1	Impact of output (output gap in relation to potential output) on inflation rate
$\vartheta$	0.1	Impact of foreign inflation on domestic inflation rate
$\varepsilon$	0.5	Adaptation of current inflation rate to previous period’s inflation rate
$\omega$	0.5	Impact of output gap in relation to potential output on unemployment rate (Okun’s Law coefficient)

The model is essentially a Keynesian demand-side model with an income-expenditure Eq. (2) and an expectations-augmented Phillips curve (9) with adaptive expectations (11). The nominal interest rate is influenced by the prime rate of the central bank but also by its lagged value and the government budget deficit, the latter effect being due to a risk premium for government bonds. Including the lagged values of the dependent variables in Eqs. (2) and (6) can be interpreted as applying the Koyck transformation, with a geometric decline of the effect of the independent on the dependent variables. The inflation rate depends, in addition to the expected inflation rate, positively on the output gap (a demand-side effect) and the foreign inflation rate (a supply-side effect, or generally an effect of the international transmission of inflation). The unemployment rate at $t=0$ is equal to the natural unemployment rate. Through Okun’s Law, the actual unemployment rate depends negatively on the output gap. All variables have the dimension percent or percentage points, except for output and real fiscal surplus which are measured in constant price monetary units.

## Optimal fiscal and monetary policies for a centralized policy maker

We first consider the case of a centralized policy maker where both fiscal and monetary policy are determined by the same policy maker (which, in reality, will be the government). This case is relevant for a country without an independent central bank, either without a central bank at all, in which case the government has some means to influence the interest rate directly, or with a central bank that is working as an agent of the government. In this case, the policy maker has two instruments to control the economy: the government budget (fiscal policy) and the prime rate (monetary policy).

We assume that the aim of the policy maker is to lead the economy along a stable path or to reach that path in the case of external shocks, thereby reducing both the unemployment rate and the inflation rate gaps. This is expressed by the following objective function, which, as a loss or a cost function, is to be minimized with respect to the instrument variables *G* and *R*:

14 $$\begin{aligned} J=\frac{1}{2}\sum _{t=1}^{T}\left(\frac{1}{1+\theta } \right)^t\{\alpha _{u} \hat{u}_{t}^2 + \alpha _{\pi } \hat{\pi }_{t}^2 + \alpha _{G} \hat{G}_{t}^2 + \alpha _{R} \hat{R}_{t}^2\} \end{aligned}$$

As a single policy maker, the government minimizes *J* over the entire time horizon. When the system is in its equilibrium in $t=0$ (all $\sim$ variables on their “ideal” values and hence all $\wedge$ variables equal to 0) and there are no external shocks, the optimized system will stay along its equilibrium path for the entire planning horizon *T*. The weights of the objective (endogenous target and exogenous instrument variables) variables are shown in Table 3 and the “ideal” values of the objective variables in Table 4. These assumptions imply that the (hypothetical) single policy maker wants to steer the target variables, namely the unemployment rate and the inflation rate, towards their equilibrium values and attaches equal weights to them but also has costs due to deviations of the instrument variables from their “ideal” values of 0 for *G* (a balanced budget) and 0.03 for *R* (the original and long-run prime rate). The numerical difference between the weights for *G* and *R* is due to higher average values for $\frac{\hat{G}}{\tilde{y}}$ than *R* over the time horizon and implies approximately equal weights also for $\frac{\hat{G}}{\tilde{y}}$ and *R*. The costs of budget deficits (negative values of *G*) come from their effects on the interest rate through Eq. (6) and from increasing public debt (not modelled explicitly in this model).

**Table 3:** Weights of the objective variables

$\alpha _{u}, \alpha _{\pi }$	$\alpha _{G}$	$\alpha _{R}$
5	0.01	0.1

**Table 4:** “Ideal” values of the objective variables

$\hat{\pi }$	$\hat{u}$	$\hat{G}$	$\hat{R}$
0	0	0	0

**Fig. 1:**
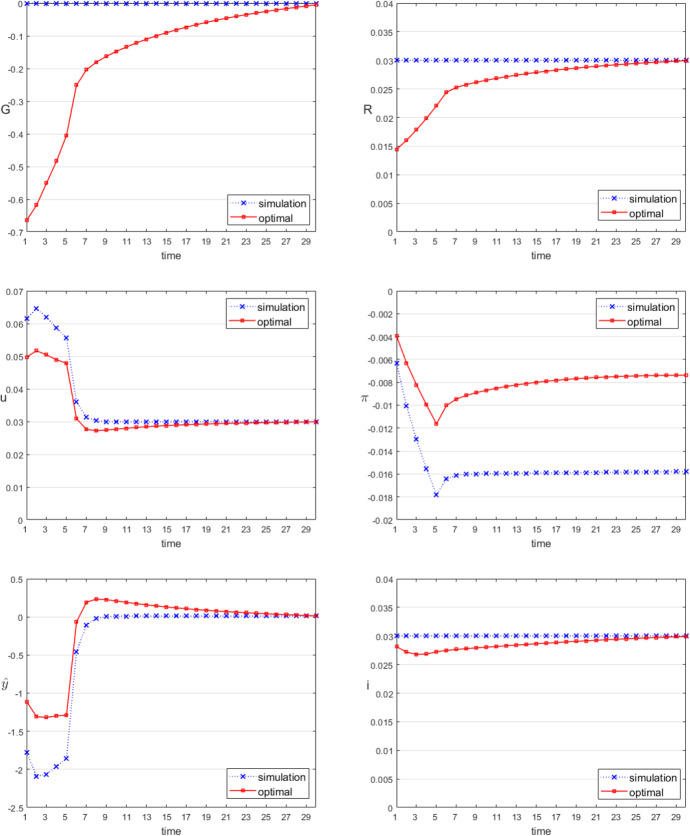
Time paths of the control and selected state variables, demand shock

We now ask what happens if an external demand shock from abroad hits the country. The shock has the values for $zd_{t}$ as given in Table 5. It is a temporary decreasing negative shock from abroad lasting five periods, measured in terms of constant price monetary units of the domestic country. It affects both target variables $\hat{y}$ and $\hat{\pi }$ negatively without policy measures (in the simulated run), which is shown in the paths named “simulation” in Fig. 1. The quadratic specification of the objective function implies that overshooting and undershooting these target variables of equal size cause the same costs for the policy maker. This may be considered questionable but is unavoidable for the symmetric objective function that is assumed here. An asymmetric objective function was investigated by Friedman (1975) but is inappropriate for the present model as it may lead to the overall instability of the entire macroeconomic system due to the assumed growth path of the “natural” output.

**Table 5:** Modelling an exogenous demand-side crisis

*t*	1	2	3	4	5	6	$\ldots$
$zd_{t}$	− 1.78	− 1.65	− 1.55	− 1.46	− 1.38	0	0

The optimal paths of the instrument variables and target variables are calculated using the OPTCON algorithm (Blueschke et al. 2021) and shown in Fig. 1 as “optimal”. For the instrument variables, these imply a government budget deficit of about 2.24 percent of the optimal natural output, and a decrease in the prime rate by 1.44 percentage points (to less than half of its “ideal” value) in the first period. Afterwards, both instruments stay below their “ideal” values but the differences decrease, first - and even during the shock period (the foreign demand recession) - quickly and later slowly until they reach zero in the last period. Both instruments act in an expansionary way, in accordance with Keynesian prescriptions for a demand shock. Their main effect is on output (aggregate demand), as shown by its deviation from the long-run or natural (and “ideal”) output, $\hat{y}$ in Fig. 1. Optimal policies result in an increase in the unemployment rate by 5 percentage points as compared to 6 percentage points in the scenario without policy intervention during the shock period, but it returns quickly to the natural rate afterwards. The nominal market interest rate decreases only slightly (by a maximum of 2.3 percentage points in period 3), the optimal rate of inflation decreases continually to a deflation rate of 1.12 (1.18 in the solution without policy intervention) in period 5 before returning to a new optimal level of deflation of less than 0.8 percent (1.16 in the uncontrolled case) and the optimal real interest rate increases as a result to a new permanent value of 3.73 percent (4.58 in the uncontrolled case).

It should be noted that this Keynesian optimal policy calls for only gradual reduction of the government budget deficit and increase in the prime rate towards their steady-state values after the shock over the entire planning horizon, which has actually been observed for real policy makers. The price for this is a mild deflation and a higher real interest rate. Both effects vanish for $t \rightarrow \infty$ if there are no further shocks in the future. This is a heroic assumption when considering the fact that real economies are often subject to frequent and even multiple shocks. Therefore, one must be very cautious when policy prescriptions are derived from such Keynesian policies for a finite time horizon and only temporary shocks. In reality, planning by policy makers under frequent shocks may have to increase the weights for the instrument variables, calling for faster return to the steady state, or otherwise may make the macroeconomic system unstable (lead it away from the steady state). Optimal policies dealing with frequent or even permanent shocks will be very different than those shown here.

**Fig. 2:**
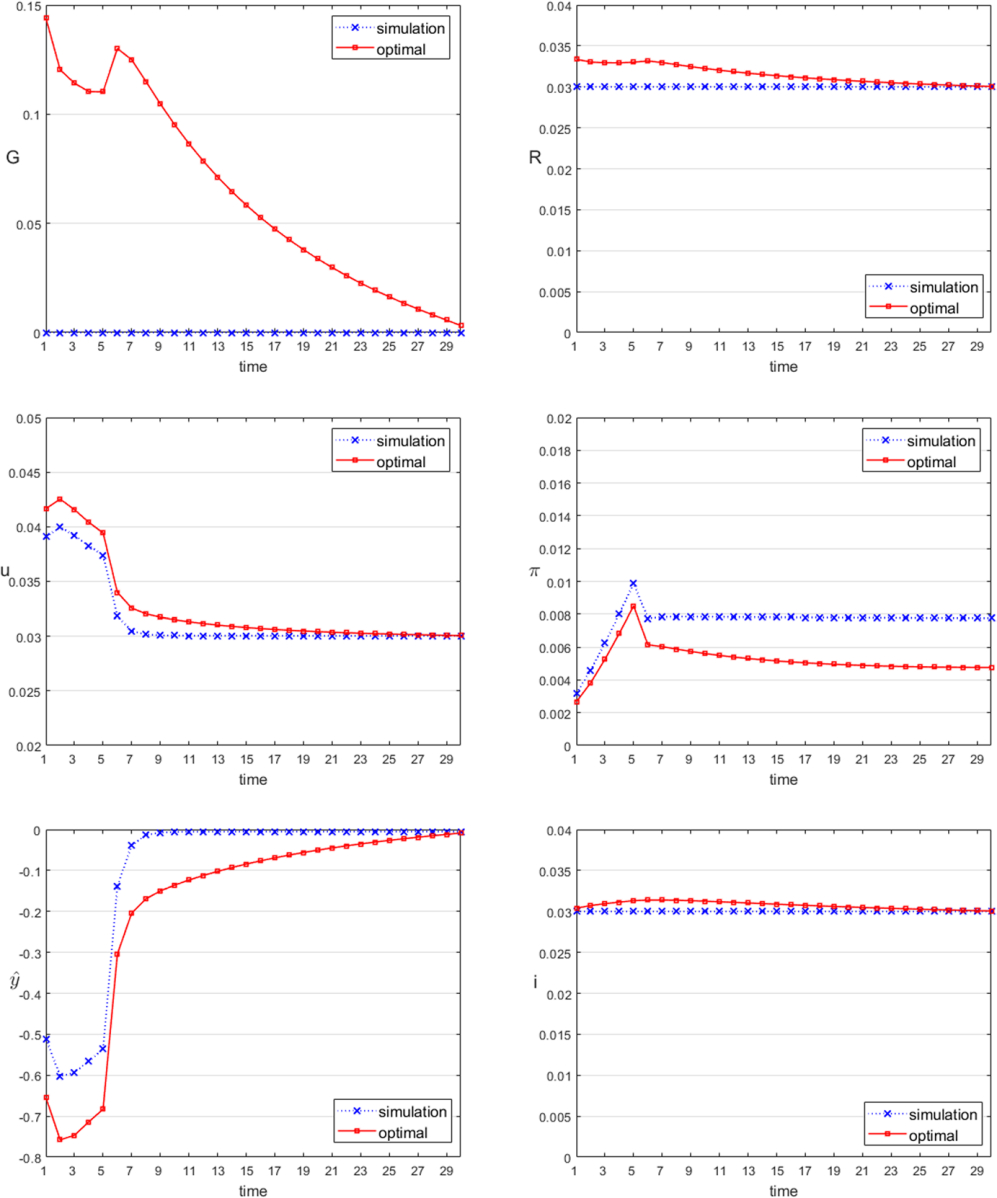
Time paths of the control and selected state variables, supply shock

Alternatively, we consider a negative supply shock, for instance, an increase in the price of a foreign factor of production (e.g., energy) or a disruption of supply chains. This implies both an increase in the foreign rate of inflation and a decrease in foreign output. The combination of these two factors leads to a decrease in domestic real output (Eq. 2) and through Eqs. (9) and (12) simultaneously to an increase in the domestic rate of inflation and rate of unemployment. In contrast to the pure demand shock, here policy makers are confronted with an unpleasant trade-off: if they act in an expansionary way, they decrease unemployment but increase inflation, if they act in a restrictive way, they decrease inflation but increase unemployment. Table 6 shows the assumed numerical values of the shock variables. Figure 2 presents the results for the uncontrolled (simulated) and optimal policies.

**Table 6:** Modelling an exogenous supply shock

*t*	1	2	3	4	5	6	$\ldots$
$\hat{y}^{ex}$	− 1.07	− 0.99	− 0.93	− 0.87	− 0.83	0	0
$\pi ^{ex}$	0.05	0.05	0.05	0.05	0.05	0	0

As expected, the supply shock results in decreases in $\hat{y}_t$ and increases in $u_t$ and $\pi _t$. The immediate effects on the unregulated and the optimal $\hat{y}_t$ are smaller than in the demand shock presented above because the decrease in $\hat{y}^{ex}$ is smaller than the shock in Table 5, the effect of which is even reinforced by the increase in $\pi _t$. Both the unemployment rate and the inflation rate increase by less than under the demand shock.

The interesting thing about the supply shock is the course of policies: increasing the budget deficit to combat increasing unemployment would increase inflation beyond the initial rise. On the other hand, a restrictive monetary policy combats inflation but increases the interest rate and thus leads to an additional decrease in output and hence increase in unemployment. In our model, optimal policies call for a restrictive course for both instruments: an increase in both the government budget surplus and the prime rate. The result is a smaller increase in inflation and a higher increase in unemployment than without active policies. However, the unemployment rate returns relatively quickly to the natural level while the inflation rate remains permanently above the steady-state level. While the effects of the shock on real variables (output and unemployment) are temporary, the price effect – though rather small – is permanent. This explains the relatively conservative policy prescription for the supply-side shock. Again, this result should be interpreted very carefully when it comes to conclusions for actual policy making for a real economy. The actual optimal policy mix for fiscal and monetary policy is a delicate decision, depending on the policy maker’s preferences between unemployment and inflation and the relative effects of the instruments on the target variables as well as the relative importance of the instruments versus the endogenous targets.

## Dynamic games between fiscal and monetary policies

In this section, we analyze the case of two different institutions being responsible for fiscal and monetary policy, the government for the former and the central bank for the latter. They have different objective functions, namely $J^{gov}$ for the government and $J^{cb}$ for the central bank:

15 $$\begin{aligned} & J^{gov}=\frac{1}{2}\sum _{t=1}^{T} \left(\frac{1}{1+\theta } \right)^t\{\alpha ^{gov}_{\pi } \hat{\pi }_{t}^2 + \alpha ^{gov}_{u} \hat{u}_{t}^2 + \alpha ^{gov}_{G} \hat{G}_{t}^2\} \end{aligned}$$

16 $$\begin{aligned} & \quad J^{cb}=\frac{1}{2}\sum _{t=1}^{T} \left(\frac{1}{1+\theta } \right)^t\{\alpha ^{cb}_{\pi } \hat{\pi }_{t}^2 + \alpha ^{cb}_{u} \hat{u}_{t}^2 + \alpha ^{cb}_{R} \hat{R}_{t}^2\} \end{aligned}$$

The instrument of the government is the budget surplus while the central bank controls the prime rate. As voters mostly punish higher unemployment more than higher inflation, we assume that the government puts a greater emphasis on the unemployment rate while the central bank gives more weight to inflation. Moreover, the players include their own control variables in the objective function as stated in Table 7. We assume the same desired paths of the objective variables as in the centralized scenario (see Table 4), with the short-run inflation gap and short-run deviation from the natural unemployment rate targeted to be zero. The weights given to the policy instruments of each player partly reflect the desire to avoid too excessive a fluctuation in these variables.

**Table 7:** Weights of the variables in the objective functions

$\alpha ^{gov}_{u}, \alpha ^{cb}_{\pi }$	$\alpha ^{gov}_{\pi }, \alpha ^{cb}_{u}$	$\alpha ^{gov}_{G}$	$\alpha ^{cb}_{R}$
10	1	0.01	0.1

We determine two solutions for the dynamic game specified by the assumptions made here. A noncooperative solution assumes strategic behaviour by both players (policy makers) without a commitment on either side and without cooperation between them. The Markov-perfect (strongly time consistent) feedback Nash equilibrium is the appropriate tool for such a situation. In contrast, a Pareto optimal solution assumes binding cooperative arrangements between the players. Pareto optimal solutions are generally not equilibria as all players have an incentive to deviate from the optimal solution. Strong institutional rules have to be established if one wants to implement cooperation between the players. In our model, for the Pareto optimal (efficient) solution, a strong binding mechanism between the government and the central bank must exist, for instance, a constitutional requirement that each institution has to act in accordance with the other one. For the cooperative Pareto scenario, the joint objective function is given by the equally weighted sum of the two objective functions in these variables:

17 $$\begin{aligned} J=0.5J^{gov} + 0.5J^{cb}. \end{aligned}$$

**Fig. 3:**
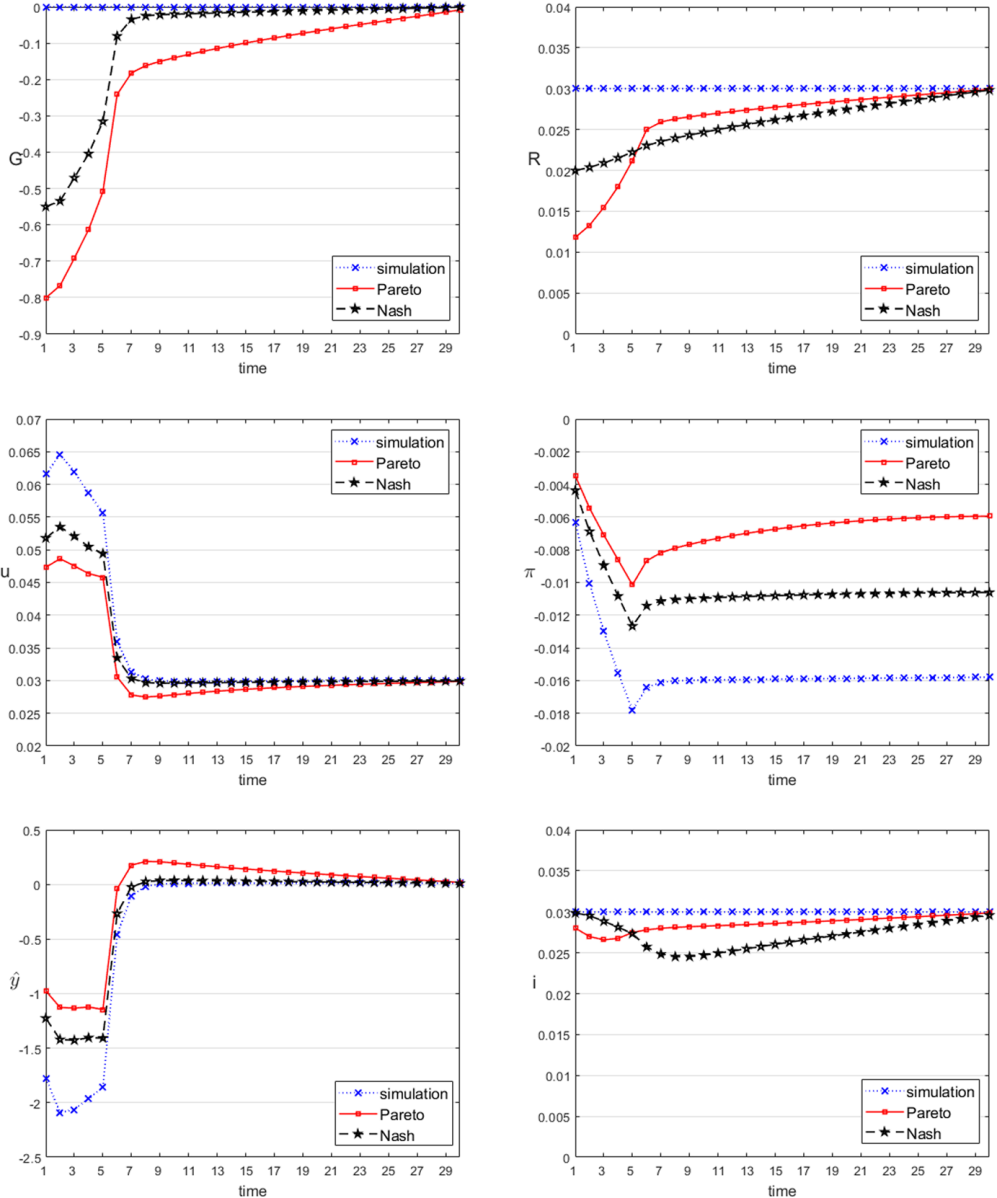
Time paths of the control and selected state variables, demand shock

Solutions of the nonlinear dynamic tracking game problem cannot be obtained analytically but have to be numerically approximated using the OPTGAME algorithm (Behrens and Neck 2015; Blueschke et al. 2013). This algorithm allows us to find approximations to cooperative (Pareto optimal) and non-cooperative Markov perfect (feedback) Nash equilibrium solutions of the game.

Consider first the game solutions for the demand shock from the previous section (see Table 5). The time paths of the instruments and the resulting target variables for both solution concepts are shown in Fig. 3. While both solution concepts call for expansionary policies, there are also clear differences between the noncooperative (denoted by Nash) and the Pareto solutions. In the Pareto solution, fiscal policy is definitely more active, producing higher budget deficits than in the Nash solution. The prime rate of the central bank starts from lower values in the Pareto solution than in the Nash one, but after period 5 the ranking is reversed and in the Pareto solution monetary policy returns more quickly to the final steady state than in the Nash solution. The cooperative solution is generally more active than the noncooperative one, as can be seen from the development of the variable $\hat{y}$, which is always closer to its desired value of 0 in the Pareto solution than in the Nash one. Both paths are closer to 0 than the uncontrolled solution, which means that active stabilization policies have a strong influence on output. Both target variables show paths that are definitely better than the uncontrolled ones, the cooperative solution being definitely better than the noncooperative one. This order can also be seen when comparing the joint objective function values (*J*) in both solution concepts: it is 0.051 in the uncontrolled solution, 0.029 in the Nash solution, and 0.026 in the Pareto solution. Thus, there is a stronger gain from active policies over inactive ones, while the gain from cooperation is much smaller than noncooperation but still positive. Demand shocks therefore call for active policies by both fiscal and monetary policy makers.

**Fig. 4:**
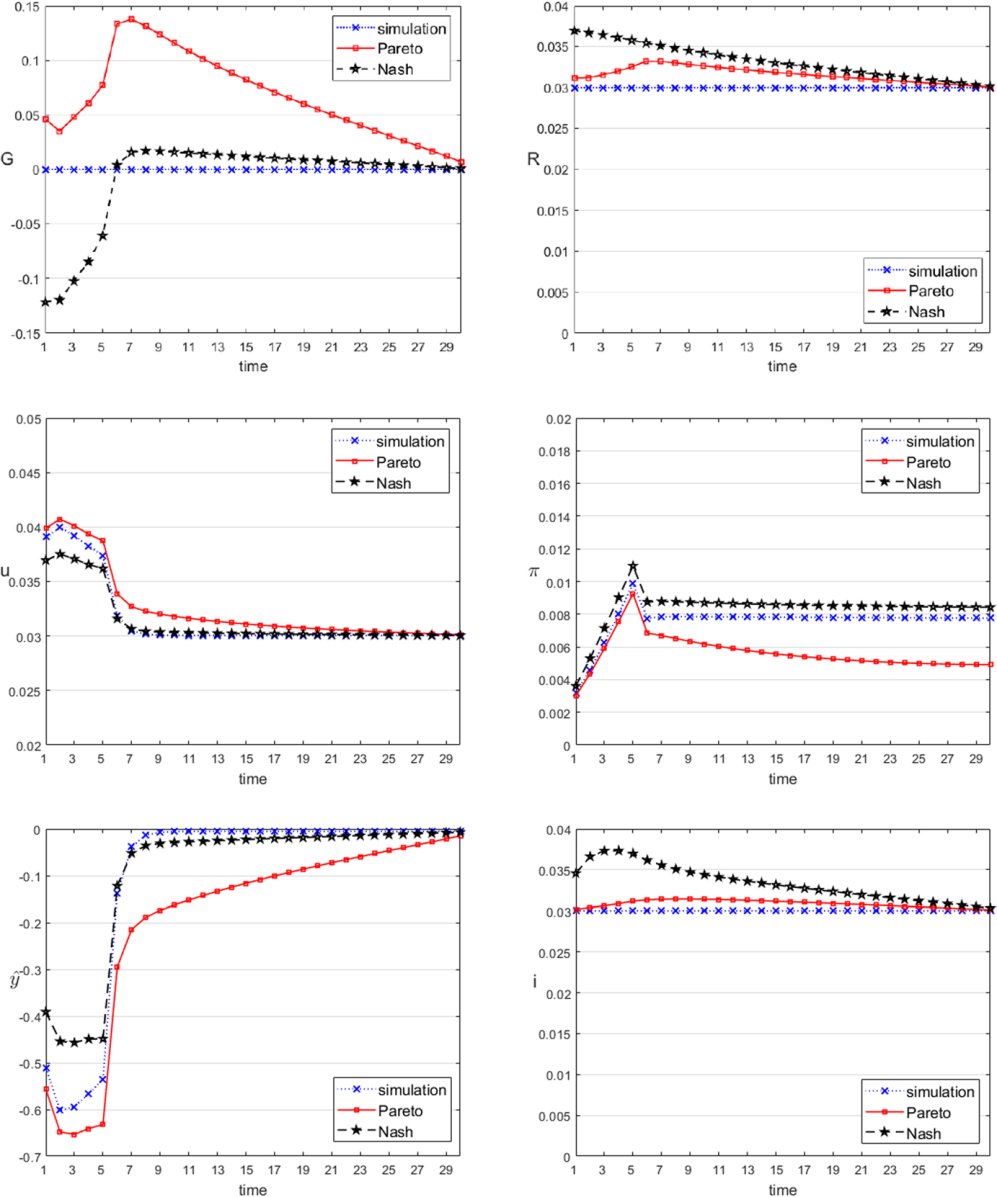
Time paths of the control and selected state variables, supply shock

The situation is different for a supply shock (see Table 6), as can be seen from Fig. 4. In this case, the noncooperative solution gives an even higher value (0.009) for the joint objective function than the uncontrolled solution (0.008) while both are worse than in the cooperative case (0.007). Especially the costs for the central bank are highest in the Nash game. This comes from the following policy mix: in the Pareto case, the government follows a restrictive policy (budget surpluses), while in the Nash solution it acts in an expansionary way (budget deficits). The central bank acts, as expected from its preferences, restrictively to combat inflation in both solution concepts, and more so in the Nash case than in the Pareto one. This results in lower values of inflation in the Pareto case than in the Nash one, and considerably so towards the end of the planning period, at the expense of only very slightly higher unemployment. At least in this particular example, the central bank and the government act in parallel in the cooperative agreement (as a single policy maker would do) while in the noncooperative equilibrium, they are at cross purposes. The Pareto solution is qualitatively very similar to the centralized optimum, as can be expected from the choice of the joint objective function (17); with unequal weights for the two policy makers (different power of the players in the cooperation), different Pareto optimal solutions will emerge.

## Concluding remarks

In this paper, we modified and extended an economic model developed by Leitmann and Wan by introducing a few additional macroeconomic hypotheses. From a methodological point of view, we combined the long-run growth model by Leitmann and Wan with a short-run macroeconomic model of stabilization policies, formulated in terms of deviations from the growth path. We determined optimal policies for a policy maker in charge of fiscal and monetary policies for a demand and a supply shock affecting the unemployment rate and inflation rate in the model. We also solved a strategic dynamic game for a version of the model where two policy makers, a government and a central bank, have their own different instruments and different preferences. We found that a cooperative Pareto solution is generally superior to a noncooperative Nash equilibrium for the game. Except for a case of noncooperation, active policies were found to be superior to no action, although the case for active policies is not as strong for a supply shock as for a demand shock. For actual policy makers, a conclusion from this analysis would be to aim at institutional rules to ensure that government and central bank envisage at least some degree of cooperation between them.

Of course, many topics remain open for further research. More simulations and optimization experiments are required to arrive at stronger conclusions. For example, uncertainty was absent from our agenda and either stochastic or other (including Leitmann’s) versions of uncertainty should be examined. The model needs further extensions and modifications as well as additional analyses, such as introducing alternative hypotheses about (especially forward-looking) expectations. In any case, it is remarkable that a model from nearly fifty years ago still has relevance today and can inspire new developments.

## Footnotes

1 We also solved the model with $T=60$. $\tilde{y}_T$ increases in this case to 87.75, but all other state and control variables take values which are close to the steady-state solution. Thus, the following results are robust to the choice of *T* as long as this is sufficiently large, which is the case for $T=30$.
